# Risk factors for central nervous system tumors in children: New findings from a case-control study

**DOI:** 10.1371/journal.pone.0171881

**Published:** 2017-02-17

**Authors:** Rebeca Ramis, Ibon Tamayo-Uria, Diana Gómez-Barroso, Gonzalo López-Abente, Antonio Morales-Piga, Elena Pardo Romaguera, Nuria Aragonés, Javier García-Pérez

**Affiliations:** 1 Cancer and Environmental Epidemiology Unit, National Center for Epidemiology, Carlos III Institute of Health, Madrid, Spain; 2 Consortium for Biomedical Research in Epidemiology & Public Health (CIBER Epidemiología y Salud Pública—CIBERESP), Madrid, Spain; 3 Centre for Research in Environmental Epidemiology (CREAL), Barcelona, Spain; 4 Universitat Pompeu Fabra (UPF), Barcelona, Spain; 5 National Center for Epidemiology, Carlos III Institute of Health, Madrid, Spain; 6 Rare Disease Research Institute (IIER), Carlos III Institute of Health, Madrid, Spain; 7 Consortium for Biomedical Research in Rare Diseases (CIBERER), Madrid, Spain; 8 Spanish Registry of Childhood Tumors (RETI-SEHOP), University of Valencia, Valencia, Spain; North Carolina State University, UNITED STATES

## Abstract

**Background:**

Central nervous system tumors (CNS) are the most frequent solid tumor in children. Causes of CNS tumors are mainly unknown and only 5% of the cases can be explained by genetic predisposition. We studied the effects of environmental exposure on the incidence of CNS tumors in children by subtype, according to exposure to industrial and/or urban environment, exposure to crops and according to socio-economic status of the child.

**Methods:**

We carried out a population-based case-control study of CNS tumors in Spain, covering 714 incident cases collected from the Spanish Registry of Childhood Tumors (period 1996–2011) and 4284 controls, individually matched by year of birth, sex, and autonomous region of residence. We built a covariate to approximate the exposure to industrial and/or urban environment and a covariate for the exposure to crops (GCI) using the coordinates of the home addresses of the children. We used the 2001 Census to obtain information about socio-economic status (SES). We fitted logistic regression models to estimate odds ratios (ORs) and 95% confidence intervals (95%CIs).

**Results:**

The results for all CNS tumors showed an excess risk (OR = 1.37; 95%CI = 1.09–1.73) for SES, i.e., children living in the least deprived areas had 37% more risk of CNS tumor than children living in the most deprived areas. For GCI, an increase of 10% in crop surface in the 1-km buffer around the residence implied an increase of 22% in the OR (OR = 1.22; 95%CI = 1.15–1.29). Children living in the intersection of industrial and urban areas could have a greater risk of CNS tumors than children who live outside these areas (OR = 1.20; 95%CI = 0.82–1.77). Living in urban areas (OR = 0.90; 95%CI = 0.65–1.24) or industrial areas (OR = 0.96; 95%CI = 0.81–1.77) did not seem to increase the risk for all CNS tumors together. By subtype, Astrocytomas, Intracranial and intraspinal embryonal tumors, and other gliomas showed similar results.

**Conclusion:**

Our results suggest that higher socioeconomic status and exposure to crops could increase the risk of CNS tumors in children.

## Background

Central nervous system (CNS) tumors in children are a variety of distinct histological tumors subtypes mostly found in the brain. After leukemia, CNS tumors are the second most common cancer in children and the most frequent solid tumor [1]. These tumors account for 20% of cancers in children [2], with an incidence slightly higher in boys than in girls, varying within the subtypes [3]. In addition, CNS tumors have the highest mortality among all forms of infant cancer [1]

As with other tumors, the current consensus is that CNS tumors are a consequence of cumulative genetic alterations which interfere with the normal functioning of cell mechanisms. These alterations may be in part, or entirely, inherited but also chemical, physical or biological agents that damage DNA could work as carcinogens. Causes of CNS tumors are mainly unknown and only 5% of the cases may be explained by genetic predisposition, mainly associated with established familial cancer syndromes such as neurofibromatosis type I and II, tuberous sclerosis, Li–Fraumeni, nevoid basal cell carcinoma, hereditary retinoblastoma, Rubinstein–Taybi, and others [1,4].

As to potential environmental causative agents, the only well-established risk factor is ionizing radiation [5]. Radiofrequency electromagnetic fields have recently been of considerable concern, and in 2013 a second evaluation of their carcinogenic hazard to humans was published by IARC [6]. In this volume, radiofrequency electromagnetic fields were classified as possibly carcinogenic to humans (group 2B). The topic of mobile phone use as a risk factor for CNS tumors in adults is dealt with in detail in a further National Radiological Protection Board report [7]. Their evaluation of five case-control studies and two cohort studies concluded there was no convincing evidence of a raised risk of CNS tumours in relation to mobile phone use–particularly for short induction periods–but a need for further studies related to tumors with long induction periods was noted. An acknowledgment was made of the limitations in the currently available studies with respect to length of use and the imprecision of exposure measurement. The interest of improving the evidence on the safety of mobile phone use has been reflected in the development of new studies, to address the risk associated with mobile phone use among children and adolescents [7]; and also among adults [8]

In the past few decades there have been studies on viruses and infections but no conclusive evidence has been shown [4,5,9]. Harmful chemicals, such as N-nitroso compounds, polycyclic aromatic hydrocarbons or metals (e.g. cadmium or lead) have motivated various studies and their results have highlighted some type of association with CNS tumors, but again no conclusive links have been established [4,5]. That said, we should not forget the difficulties of finding conclusive evidence when CNS tumors in children are studied, for instance: small numbers of cases, tumor heterogeneity, unknown latency period or period of vulnerability of the brain to these compounds, recall difficulties, and other methodological issues.

Specific characteristics of the environment in which the child lives, such as proximity to industrial areas, urban areas and crops, could play an important part in the incidence of CNS tumors in children [10]. A Taiwanese study found an association between petrochemical air pollution and brain cancer in under 29 year olds [11], a retrospective cohort study in Great Britain found an excess of solid tumors close to a variety of industries [12], and a case-control study in the USA found increased risk of brain cancer associated with residential proximity to factories during pregnancy [13]. For urban environments, several traffic-related studies in USA did not show conclusive results for CNS tumors in children [14,15]; however, a more recent study suggested positive associations between hazardous air pollutants and incidence of astrocytoma [16]. With regard to pesticide exposure, a meta-analysis showed excess of risk for brain cancer–influenced by father’s exposure [17]. Nonetheless, other studies did not find excess of risk [18,19]. In addition to the living environment, the socioeconomic status of the family could play a role in CNS tumor incidence. Some studies addressed these questions but their findings were inconclusive [20–22]

In this paper we studied the effects of various environmental exposures on the risk of CNS tumors in children. In particular, we studied the effect of exposure to industrial and/or urban environment as proxies of exposure to air pollution, exposure to crops as proxy of exposure to pesticides and the socio-economic status of the child.

## Methods

### Data

This paper is part of a population-based case-control study which aims to analyze the effect of environmental risk factors over childhood cancer using the geographic locations of the cases and controls in Spain. Specific details for the design of the study can be found in the previous papers from the project [23,24]. For the reader’s convenience, a summary of the design can be found below.

**Fig 1:**
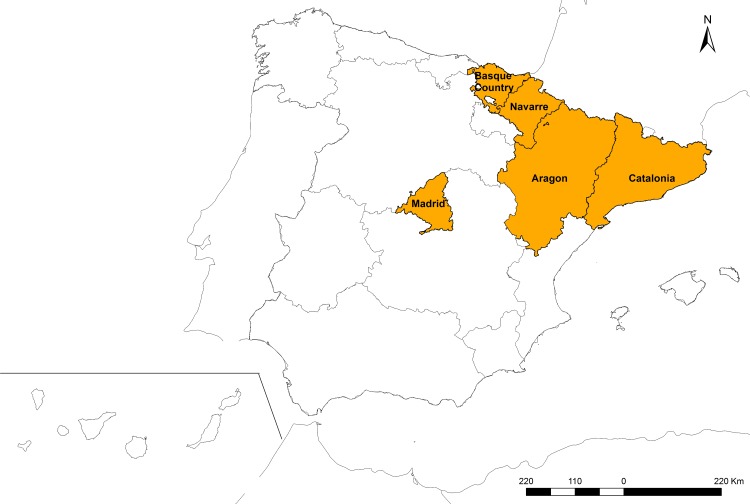
Map of the studied regions.

For the study we used data from children aged 0 to 14 with diagnosed central nervous system neoplasms, Group III from the International Classification of Childhood Cancer, Third Edition (ICCC-3) [25]. Cases were classified by subgroup: IIIa) Ependymomas and choroid plexus tumor, IIIb) Astrocytomas, IIIc) Intracranial and intraspinal embryonal tumors (IIET), IIId) Other gliomas, IIIe) Other specified intracranial and intraspinal neoplasms (other specified), IIIf) Unspecified intracranial and intraspinal neoplasms (unspecified). Incidence cases were registered by the Spanish Registry of Childhood Tumors (RETI-SEHOP). RETI-SEHOP collects information from cases of childhood cancer from hospital pediatric oncology units over all Spain [26]. The studied period went from 1996 to 2011. As a control group we used a random sample from the population at risk extracted from the Birth Registry of the National Statistics Institute (Instituto Nacional de Estadística, INE). Controls were individually matched to cases by sex, year of birth and autonomous region of residence, in a ratio of 6:1. We analyzed five Spanish regions: the Autonomous Region of Madrid, the Basque country, Aragon, Navarre, and Catalonia. Fig 1 shows the exact location of these regions within Spain.

We geocoded the home addresses of the cases at the moment of diagnosis (included in the RETI-SEHOP), and the mother’s the home address at birth for the controls (included in the Birth Registry of the INE). We successfully validated 98% of addresses for the cases. The remaining 2% were fairly uniformly distributed across the different regions and, therefore, we did not think the data were biased in this sense. Only 2% of controls did not have valid coordinates. Given that the number of failures was small, we decided to select more controls to replace the 2%, and we geocoded and validated this last group to finish with 6 controls with valid coordinates for each case. Using the entries for the Birth Registry, we managed to recover the address at birth for 332 cases; we used a matching strategy to find cases with the same address on the birth certificate and in the RETI-SEHOP.

#### Industrial-urban environment exposure

We built an exposure variable to account for the kind of environment in which the child lived using the geographical coordinates of the residences. We categorized this variable as industrial, urban, industrial-urban (intersection) and rural, as we did in a previous paper from the project [27]. We classified every child as: a) residence in an *“industrial area”*, defined in terms of proximity to industrial facilities (Table A in S1 File: list of industrial groups) according to a predefined distance ‘D’; b) resident in an *“urban area”*, taking the areas defined according to the size of the municipality; c) resident in an intersection between industrial and urban areas (*“both”*); and, d) resident within a rural area, *“reference area”*, consisting of zones in which children had no industry within 5 km of their residences and which were far from urban areas. We ran a sensitivity analysis to choose the distance for the industrial area by fitting the models for the following distances ‘D’: 5, 4, 3, 2.5, 2, 1.5, and 1 km (Figure A in S1 File).

#### Socioeconomic status

To study the relationship between the socioeconomic status of the child and risk of CNS tumors we used data from the 2001 Census [28] because we did not have individualized information for the children in our study. This information was at census tract level; therefore, we assigned the information of the corresponding census tract to every child. We used the variable denominated “socioeconomic condition” (SES), which was based on the occupation of the head of the family. This variable ranged from 0.46 to 1.57; lower values were assigned to worse socio-economic status and higher values to better socioeconomic status. We computed the quartiles of the variable to introduce it into the model.

#### Crop exposure

To assess the effect of exposure to crops on childhood cancer risk we built a specific index that was presented in a previous paper [29]. This index accounts for the percentage of cultivated land in a 1-km buffer around the child’s residence. The general index, Global Crop Index (GCI), includes 6 subcategories of crops: arable land or permanently irrigated land (Irrigated), rice fields (Rice), vineyards (Vineyards), fruit trees and berry plantations (Fruits), olives groves (Olives), and heterogeneous agricultural areas (Heterogeneous). For the present analysis we also computed the percentage of cultivated land for every individual crop subcategory. The information used to build the index came from Corine Land Cover 2006 [30].

### Statistical analysis

As just mentioned, before fitting the final models we ran a sensitivity analysis to choose the distance D that defined the end of the exposure area for industrial pollution. Once we established distance D, we estimated the Odds Ratio and 95% confidence intervals (95%CIs) associated with the exposure covariates by fitting mixed multiple unconditional logistic regression models. We included as categorical variables the industrial-urban environment and the SES, and the GCI was a continuous variable. We included the matching factors sex, year of birth and region (as a random effect). We repeated the analysis by CNS tumor subgroup and we also analyzed the crop subcategories individually. We used the group of cases with the same address at the time of birth and at the time of diagnosis to perform a sensitivity analysis to evaluate potential bias introduced by the diagnosis addresses. Finally we performed another sensitivity analysis to assess potential regional differences. We used R library Lmer4 [31] for statistical analysis and GIS to build the indexes.

## Results

After geocoding and validation, we had 714 cases of CNS tumors and, therefore, we matched 6 times the number of controls, 4284. Table 1 shows the distribution of cases by subgroup, where Astrocytomas comprised the main subgroup with 36% of all cases, followed by Intracranial and intraspinal embryonal tumors, with 24% of all cases, the remaining 4 subgroups had less than 14% of the cases each. The incidence age mean varied from 3.4 to 6.4 years old, and by sex, two subgroups showed higher incidence in girls and three subgroups showed higher incidence in boys. We were able to find 332 cases with the same address at the time of birth and at the time of diagnosis and the distribution by tumor subgroup was very similar to the full cases dataset. Table 2 shows the distribution of the cases by region.

**Table 1:** Distribution of cases by subgroup, incidence age mean and number of cases by sex.

SubGroup	Cases (%)	Incidence age mean (years)	Boys	Girls	Cases with same address at birth and at diagnosis
Ependymomas and choroid plexus tumor	94 (13%)	3.4	55	39	55 (17%)
Astrocytomas	258 (36%)	5.4	129	129	112 (34%)
Intracranial and intraspinal embryonal tumors (IIET)	171 (24%)	4.2	102	69	78 (23%)
Other gliomas	88 (12%)	5.5	38	50	40 (12%)
Other specified intracranial & intraspinal neoplasms	72 (10%)	6.4	46	26	28 (8%)
Unspecified intracranial and intraspinal neoplasms	31 (4%)	5.6	14	17	19 (6%)
Total CNS	714	4.9	384	330	332

**Table 2:** Distribution of the cases by region.

Region	Total	Boys	Girls
Aragon	52	30	22
Catalonia	343	178	165
Madrid	201	111	90
Navarre	35	23	12
Basque Country	83	42	41

The results for the sensitivity analysis over the distance ‘D’ showed that the trend for the estimated OR of exposure to urban areas had a change point at 2km (results in the appendix Figure A in S1 File). Consequently, we established 2 km as the maximum distance for the exposure to industries for the forthcoming analyses. This created a fith category in the “Industrial, urban and rural status” variable that included the zones out from urban areas and between 2km and 5km to the nearest factory (This category included 162 cases and 1014 controls, the results were not showed)

Table 3 shows the results for the model including the covariates industrial-urban environment, SES and GCI. The ORs for GCI showed the change in the odds for an increase of 10% in the GCI. The OR for SES is the one that compares children in the fourth quantile, better condition, against children in the first quartile, worse condition (Results for the evolution of the OR for the SES within the four quantiles are in the Figure B in S1 File). The results for Total CNS tumors showed a statistically significant increased odds for SES with an OR of 1.37 (95%CI = 1.09–1.73), meaning that children who lived in the least deprived areas had 37% more risk of developing a CNS tumor than children living in the most deprived areas. OR for GCI showed statistically significant increased odds (OR = 1.22; 95%CI = 1.15–1.29); an increase of 10% of the cultivated land in the 1-km buffer around the child’s residence implied an increase of 22% in the OR. Those children who lived in the intersection of industrial and urban areas had more probability of developing CNS tumors compared to those who lived outside of the urban and industrial areas; however, this OR was not statistically significant (OR = 1.20; 95%CI = 0.82–1.77). Finally, living in urban (OR = 0.90) or industrial (OR = 0.96) areas did not seem to increase the risk for Total CNS tumors.

**Table 3:** Number of controls and cases and % for each category of each covariate; OR and 95%CI for the covariates industrial-urban environment (IUR), SES and GCI, for the Total of CNS and for each CNS tumor subgroup. ORs for SES show the ORs of children in the 2nd, 3rd and 4th quartile vs children in the lower quartile. OR for GCI show the OR per increase of 10% in the index. The IUR variable has a fifth category with 71 cases and 1014 controls located out from urban areas and between 2km and 5km to the nearest factory (ORs for this category are not showed).

			CNS			Ependymomas			Astrocytomas			IIET	
Exposure variable	Controls (%)	Cases (%)	OR	95%CI	Cases (%)	OR	95%CI	Cases (%)	OR	95%CI	Cases (%)	OR	95%CI
**IURS Rural(Reference)**	421 (10)	88 (12)	1	-	9 (9)	1	-	31 (12)	1	-	23 (13)	1	-
**IURS Industrial (2km)**	1837 (43)	301 (42)	0.96	(0.73,1.26)	37 (40)	1.04	(0.48,2.22)	109 (42)	1.07	(0.69,1.66)	72 (42)	0.9	(0.51,1.42)
**IURS Intersection**	288 (7)	54 (7)	1.2	(0.82,1.77)	10 (10)	1.78	(0.69,4.61)	15 (5)	1.08	(0.56,2.08)	12 (7)	1	(0.45,2.01)
**IURS Urban**	695 (16)	109 (15)	0.9	(0.65,1.24)	15 (16)	1.12	(0.47,2.68)	44 (17)	1.11	(0.67,1.83)	23 (13)	0.7	(0.39,1.38)
**SES (1Q reference)**	1135 (26)	152 (21)	1	-	25 (27)	1	-	44 (17)	1	-	28 (22)	1	-
**SES (2Q vs 1Q)**	1045 (24)	173 (24)	0.97	(0.76,1.24)	26 (28)	0.86	(0.63,1.17)	64 (25)	0.82	(0.62,1.09)	44 (26)	0.85	(0.64,1.13)
**SES (3Q vs 1Q)**	1105 (26)	160 (22)	1.01	(0.80,1.28)	15 (16)	1.07	(0.80,1.44)	63 (24)	0.88	(0.67,1.17)	42 (24)	1.08	(0.82,1.42)
**SES (4Q vs 1Q)**	1135 (26)	229 (32)	**1.37**	**(1.09,1.73)**	28s (28)	1.01	(0.57,1.77)	87s (34)	**1.78**	**(1.21,2.61)**	47 s (27)	1.1	(0.71,1.79)
**Global Crop Index (GCI)**	574^c^ (14)	180 ^c^ (25)	**1.22**	**(1.15,1.29)**	22 ^c^ (23)	1.14	(0.98,1.32)	71 ^c^ (27)	**1.27**	**(1.17,1.37)**	43 ^c^ (25)	**1.2**	**(1.07,1.30)**
			**Other gliomas**			**Other specified**			**Unspecified**				
**Exposure variable**	**Controls (%)**	**Cases (%)**	**OR**	**95%CI**	**Cases (%)**	**OR**	**95%CI**	**Cases (%)**	**OR**	**95%CI**			
**IURS Rural(Reference)**	421 (10)	10 (13)	1	-	13 (18)	1	-	2 (6)	1	-			
**IURS Industrial (2km)**	1837 (43)	42 (48)	1.29	(0.62,2.69)	30 (41)	0.62	(0.31,1.24)	11 (35)	1.36	(0.29,6.39)			
**IURS Intersection**	288 (7)	7 (7)	1.56	(0.56,4.33)	7 (9)	0.94	(0.36,2.47)	3 (10)	2.44	(0.38,15.48)			
**IURS Urban**	695 (16)	12 (13)	0.88	(0.36,2.16)	6 (8)	0.27	(0.12,0.75)	9 (29)	2.79	(0.57,13.64)			
**SES (1Q reference)**	1135 (26)	17 (19)	1	-	21 (29)	1	-	31 (23)	1	-			
**SES (2Q vs 1Q)**	1045 (24)	13 (15)	0.73	(0.53,1.00)	8 (11)	0.79	(0.58,1.09)	5 (16)	0.79	(0.57,1.09)			
**SES (3Q vs 1Q)**	1105 (26)	29 (33)	0.98	(0.73,1.31)	15 (20)	0.97	(0.71,1.31)	9 (30)	1	(0.74,1.36)			
**SES (4Q vs 1Q)**	1135 (26)	29 (33)	1.7	(0.91,3.17)	28 (39)	1.33	(0.73,2.39)	10s (32)	1.11	(0.41,3.05)			
**Global Crop Index (GCI)**	574 ^c^ (14)	24 ^c^ (27)	**1.21**	**(1.11,1.39)**	16 ^c^ (22)	1.11	(0.93,1.30)	4 ^c^ (13)	1.04	(0.73,1.50)			

^c^ number of children with >0 in the GCI variable.

The analyses by CNS tumor subgroups are also shown in Table 3. Ependymomas seemed to be associated with all variables; however, no estimated OR was statistically significant. The same occurred with astrocytomas and all variables showed excess risks too, but in this case, the ORs for SES and GCI were statistically significant. For Intracranial and intraspinal embryonal tumors (IIET), the covariates SES and GCI showed increased odds, but only the OR for GCI was statistically significant.

Table 4 shows the results for the group of cases that had the same address at the time of birth and at the time of diagnosis. The results showed very similar estimated ORs for this subgroup of cases when compared to the full group. We did not have enough cases for the Unspecified intracranial and intraspinal neoplasms to estimate the effect of industrial and urban environments.

**Table 4:** Results for the sensitivity analysis, cases with the same address at birth and at diagnosis. Number of controls and cases and % for each category of each covariate; OR and 95%CI for the covariates industrial-urban environment (IUR), SES and GCI, for the Total of CNS and for each CNS tumor subgroup. ORs for SES show the ORs of children in the 2nd, 3rd and 4th quartile vs children in the lower quartile. OR for GCI show the OR per increase of 10% in the index. The IUR variable has a fifth category with 71 cases and 1014 controls located out from urban areas and between 2km and 5km to the nearest factory (ORs for this category are not showed).

			CNS			Ependymomas			Astrocytomas			IIET	
Exposure variable	Controls (%)	Cases (%)	OR	95%CI	Cases (%)	OR	95%CI	Cases (%)	OR	95%CI	Cases (%)	OR	95%CI
**IURS Rural(Reference)**	421 (10)	39 (12)	1	-	7 (13)	1	-	17 (15)	1	-	10 (13)	1	-
**IURS Industrial (2km)**	1837 (43)	151 (45)	1.1	(0.75,1.61)	22 (40)	0.76	(0.31,1.86)	50 (45)	0.88	(0.49,1.58)	35 (45)	1	(0.48,2.11)
**IURS Intersection**	288 (7)	29 (9)	1.47	(0.87,2.49)	4 (7)	0.87	(0.24,3.13)	8 (8)	1.05	(0.43,2.55)	5 (6)	0.98	(0.32,3.01)
**IURS Urban**	695 (16)	42 (13)	0.79	(0.49,1.27)	6 (11)	0.64	(0.20,2.00)	17 (15)	0.75	(0.37,1.54)	8 (10)	0.58	(0.22,1.55)
**SES (1Q reference)**	1071 (25)	67 (19)	1	**-**	14 (25)	1	-	14 (12)	1	**-**	17 (22)	1	-
**SES (2Q vs 1Q)**	1071 (25)	82 (25)	1.15	(0.82,1.61)	20 (36)	1.3	(0.65,2.59)	31 (28)	**2.11**	**(1.11,3.99)**	18 (23)	0.97	(0.50,1.91)
**SES (3Q vs 1Q)**	1071 (25)	85 (26)	1.16	(0.83,1.63)	10 (18)	0.61	(0.27,1.38)	31 (28)	**2.01**	**(1.06,3.83)**	19 (24)	0.98	(0.50,1.89)
**SES (4Q vs 1Q)**	1071 (25)	98 (30)	**1.43**	**(1.03,2.00)**	11 (20)	0.7	(0.31,1.57)	36 (32)	**2.46**	**(1.31,4.71)**	24 (31)	1.37	(0.72,2.61)
**Global Crop Index (GCI)**	574^c^ (14)	92^c^ (28)	**1.17**	**(1.09,1.26)**	18^c^ (33)	**1.19**	**(1.12,1.27)**	33^c^ (29)	**1.2**	**(1.12,1.28)**	21^c^ (27)	**1.2**	**(1.12,1.28)**
			**Other gliomas**			**Other specified**			**Unspecified**				
**Exposure variable**	**Controls (%)**	**Cases (%)**	**OR**	**95%CI**	**Cases (%)**	**OR**	**95%CI**	**Cases (%)**	**OR**	**95%CI**			
**IURS Rural(Reference)**	421 (10)	3 (8)	1	-	2 (7)	1	-	0 (0)	1	-			
**IURS Industrial (2km)**	1837 (43)	23 (58)	**4.49**	**(1.04,19.39)**	12 (43)	2.05	(0.42,9.94)	9 (47)	-	-			
**IURS Intersection**	288 (7)	6 (15)	0.98	(0.21,4.65)	3 (11)	3.31	(0.50,22.15)	3 (16)	-	-			
**IURS Urban**	695 (16)	4 (10)	1.9	(0.72,5.02)	3 (11)	1.15	(0.17,7.62)	4 (21)	-	-			
**SES (1Q reference)**	1071 (25)	7 (17)	1	**-**	10 (36)	1	-	5 (26)	1	**-**			
**SES (2Q vs 1Q)**	1071 (25)	6 (15)	0.86	(0.29,2.58)	3 (25)	0.27	(0.07,1.00)	4 (21)	0.81	(0.22,3.02)			
**SES (3Q vs 1Q)**	1071 (25)	16 (40)	**2.45**	**(1.00,6.06)**	3 (25)	0.27	(0.07,1.01)	6 (31)	1.24	(0.37,4.16)			
**SES (4Q vs 1Q)**	1071 (25)	11 (28)	**1.02**	**(1.00,1.04)**	12 (43)	1.25	(0.52,3.00)	4 (21)	0.79	(0.20,3.14)			
**Global Crop Index (GCI)**	574^c^ (14)	12^c^ (30)	**1.18**	**(1.10,1.26)**	7^c^ (25)	**1.17**	**(1.09,1.25)**	1^c^ (5)	**1.16**	**(1.08,1.25)**			

^c^ number of children with >0 in the GCI variable.

The results for exposure to specific types of crops are shown in Table 5. Exposure to total crops seemed to increase the risk for all subtypes of CNS tumors, with statistically significant ORs for Astrocytomas, Intracranial and intraspinal embryonal tumors and other gliomas. Exposure to irrigated crops and exposure to heterogeneous crops increased the risk for total CNS tumors and for all subtypes, with statistically significant ORs for total CNS tumors, Astrocytomas, Intracranial and intraspinal embryonal tumors (IIET) and Other gliomas. Finally, exposure to fruit crops, vineyards and olives trees showed excess of risk for almost all subtypes; however, most of the estimated ORs were not statistically significant.

**Table 5:** Results by specific crop type: OR and 95%CI by specific crop type for an increase of 10% in the crop index.

	Global Crop Index		Irrigated		Heterogeneous		Fruits		Vineyards		Olives	
SubGrupo	OR	95%CI	OR	95%CI	OR	95%CI	OR	95%CI	OR	95%CI	OR	95%CI
**CNS tumors**	**1.22**	**(1.15,1.29)**	**1.27**	**(1.16,1.38)**	**1.28**	**(1.14,1.45)**	**1.18**	**(1.04,1.34)**	1.14	(0.96,1.36)	1.25	(0.93,1.67)
**Ependymomas**	1.13	(0.97,1.32)	1.12	(0.86,1.45)	**1.32**	**(1.03,1.71)**	0.81	(0.34,1.95)	1.21	(0.85,1.72)	0.74	(0.06,9.65)
**Astrocytomas**	**1.27**	**(1.18,1.37)**	**1.35**	**(1.21,1.51)**	**1.27**	**(1.06,1.51)**	**1.19**	**(1.01,1.41)**	1.13	(0.87,1.45)	**1.47**	**(1.10,1.98)**
**PNET**	**1.19**	**(1.08,1.31)**	**1.19**	**(1.02,1.38)**	**1.28**	**(1.05,1.56)**	1.18	(0.97,1.42)	1.15	(0.87,1.51)	0.94	(0.35,2.53)
**Other gliomas**	**1.22**	**(1.07,1.38)**	**1.23**	**(1.00,1.51)**	**1.34**	**(1.07,1.71)**	1.26	(0.99,1.60)	0.61	(0.05,6.44)		
**Other specified**	1.11	(0.93,1.31)	1.14	(0.90,1.46)	1.04	(0.69,1.57)	1.13	(0.82,1.54)	1.03	(0.60,1.78)		
**Unspecified**	1.04	(0.72,1.50)			1.22	(0.69,2.15)	1.08	(0.54,2.15)	1.24	(0.70,2.20)		

The results for the sensitivity analysis per region showed that the Basque Country had differential behavior. When we fitted the models without this region ORs for GCI decreased (OR = 1.00) and SES increased (OR = 1.43).

## Discussion

The results of this study suggest that the socioeconomic status play role in the risk of Total CNS tumors;; that is to say, a better socioeconomic status seems to increase the risk for all CNS tumors and for any cancer subtype individually. Exposure to crops, as a proxy of exposure to pesticides, also seems to be associated with increases in risk. We found increased risks for all CNS tumors and for individual cancer subtypes in relation to exposure to total crops and exposure to specific types of crops. Proximity to industrial or urban areas did not seem to increase the risk; however, the interaction of these two environments suggests an increase in risk.

Two of the studied regions, the Basque Country and Navarre, have shown the highest rates of brain cancer mortality in Spain for decades [32]. Moreover, Navarre had the highest child and adolescent brain cancer mortality [33]. The sensitivity analysis regarding region showed differential behavior for the ORs when excluding the Basque Country. We should mention that very few children from this region were exposed to crops and the SES of these children was higher than the average, as this region is one of the richest in Spain. Consequently, exposure to crops should not be linked to CNS tumors for the Basque Country children. On the other hand, the higher SES of the Basque Country children did not seem to substantially influence the association between cancer risk and the SES.

The role of socioeconomic status in CNS tumor incidence has been studied previously; however, the findings are inconclusive. A British study that analyzed 567 cases registered in the course of 25 years did not find any association between incidence rates of CNS tumors and deprivation index [20]. In Norway, a cohort study of all children born in Oslo from 1967 to 2009 found high risk for astrocytoma in children of parents from lower income group [21]. A case-control study of 11,119 CNS tumors diagnosed at ages <15 years from the British National Registry of Childhood Tumours and 11,039 matched controls found increased risk in children of parents in the higher social class suggesting that occupational social class of the father may be associated with risk of some childhood CNS cancers [22]. Our results agree with this last study as we found increased risk among those children in the higher quartile of the socioeconomic status compared to those children in the lower quartile, and we also found excess risk for Total CNS tumors and for all individual cancer subtypes. The association between SES and CNS tumors was the most conclusive result in our analysis.

With regard to environmental exposure, the literature tends to support the hypothesis that exposure to pesticides could be associated with brain cancer [16,33–35]. Some studies suggest that the greatest risks are associated with household insecticide use and prenatal exposure to insecticides [35]. However, a more general conclusion for these studies is that the power of their results is limited; this could be because the methods used did not provide an ideal measure of pesticide exposure and/or the heterogeneity of the CNS tumors [36]. In our study, we could not assess direct exposure to pesticides; thus we used proximity to crops as a proxy of the real exposure. In a previous paper, we presented the proxy for exposure to crops (GCI) and we have already discussed about its efficacy as subrogate [29]. In that previous analysis, we found excess risk for many types of childhood cancer and one of them was CNS tumors; our current results also showed excess risk for three subtypes of CNS tumors: astrocytoma, Intracranial and intraspinal embryonal tumors and Other gliomas. Besides this, we found excess risk for exposure to crop subcategory, specifically exposure to irrigated crops and heterogeneous crops lands. In other words, according to our results living nearby crops, especially irrigated and heterogeneous, could be associated with an increase in the incidence of all CNS tumors in children, specifically of Astrocytoma, Intracranial and intraspinal embryonal tumors and Other gliomas. Finally, there are two studies that explore the association between exposure to nitrates in drinking water, which tends to be high in agricultural areas, and an increased risk of CNS tumors in children [37,38]. However, due to the lack of information about phytosanitary products used on crops, recall bias, we cannot hypothesize about the role of a specific chemical compound. Nevertheless the use of a geographic information systems approach to combine geocoded residential addresses with land-use maps helps to minimize this recall bias and capture residential exposure that may otherwise be unknown.

For exposure to urban or industrial environments, we did not find associations for either category separately; nevertheless, in the interaction of the two environments the results did show increased risk, though these risks were not statistically significant. These results suggested that children exposed to both urban and industrial environments could have higher risk of suffering CNS tumors than in separate environments. For exposure to industries, three previous papers from our project showed association between leukemia, neuroblastoma and renal tumors in children; and residence in the proximity of industries [24,27,39]. Some pollutants released from industries, such as PAH, cadmium or lead, have motivated some studies in relation to CNS tumors in children [5]; however, the conclusions are far from establishing links. Another matter regarding urban areas nearby industrial settlements is that their populations should suffer more deprivation, which is associated with worse health status [40], yet we should not forget that our analysis included SES and the estimated association pointed to higher SES status as a risk factor.

An important difficulty when CNS tumors are under study is the different existing classifications for brain cancer. For this study we used the classification of the “International Classification of Childhood Cancer, Third Edition” proposed by Steliarova-Foucher et al. in 2005 [25]. However, other studies used the World Health Organization (WHO) classification of CNS tumours, and the problem with this classification is that it is not always possible to classify a particular brain tumor in children, due to the complexities of individual CNS tumors. In 2007, the WHO published the Fourth Edition of the “classification of tumors of the central nervous system” that included more entities [41].

One of the limitations of this study is the non-inclusion of individual data about possible confounding factors, as socioeconomic variables or lifestyle-related factors; also we did not have information about change in residence before diagnosis and after birth for the controls. As we did not have individual data we used socioeconomic data at census tract level to include socioeconomic information in the analysis. Another limitation could be the use of a circular buffer around the home residence as a proxy of exposure (for industries and crops); this assumes an isotropic model, something that could introduce a misclassification problem, since real exposure is critically dependent on prevailing winds, geographic landforms and releases into aquifers. Nevertheless, this problem would limit the capacity to find positive results while in no way invalidating the associations found. Another important limitation is that we did not have any information about parental occupational exposures at an individual level that seems to be one of the identifiable risk factors. Finally, we should not forget that the ecological nature of part of our data could be another limitation; nonetheless, the use of ecological data to generate hypothesis when studying rare cancer can be useful to better direct research resources at emerging risk factors [42].

One of the main strengths of our study is that we obtained the same results with the subgroup of cases that had the same address at the time of birth as at the time of diagnosis. Having only the address at the time of diagnosis could induce some degree of exposure misclassification [43]. In Spain there is a low rate of local migration and according to official data, only around 1% of the child population changes their residence to a different province [28].

An additional strength of our study is the large control group. Most studies of this type have one or two controls per case [44–46]. In our study we have 6 controls per case and that gives a much more realistic image of the spatial distribution of the population at risk. A further advantage of the study is the stratification of the risk by type of crop, which provides a more exhaustive description of childhood CNS tumor risk.

## Conclusions

The findings of this study suggest that children with higher socioeconomic status could have increased risk for CNS tumors. Also, exposure to crops, particularly to irrigated crops and heterogeneous crops could also increase the risk. Finally, children living in the intersections of industrial and urban areas could have a higher risk than in separate areas. Nevertheless, further studies should be carried out the attempt to understand the relationship between these environmental factors and CNS tumors.

## Supporting information

S1 File Table A: list of industrial groups, together with their E-PRTR categories, and number of installations by industrial group and autonomous region. **Figure A**. Estimated OR for industrial exposure and urban exposure with different distances ‘D’: 5, 4, 3, 2.5, 2, 1.5, and 1 km. **Figure B**. Graph showing the evolution of the OR for SES per quantile. The graph has a line per tumor subgroup. The OR for the second and third quantile were not statistically significant different that the OR for the lower quantile, only the OR for the higher quantile was statistically significant.
(DOCX)

## Abbreviations

CNS: Central nervous system
IIET: Intracranial and intraspinal embryonal tumors
RETI-SEHOP: Spanish Registry of Childhood Tumors
GCI: Global Crop Index
SES: Socioeconomic status
ORs: Odds ratios
95%CIs: 95% confidence intervals
IARC: International Agency for Research on Cancer
