# Supplementary material for: Risk factors for central nervous system tumors in children: New findings from a case-control study
Source: PLoS One. 2017 Feb 17;12(2):e0171881. doi: 10.1371/journal.pone.0171881 (PMC5315394; doi:10.1371/journal.pone.0171881)
Supplement: S1 File — Table A: list of industrial groups, together with their E-PRTR categories, and number of installations by industrial group and autonomous region. Figure A. Estimated OR for industrial exposure and urban exposure with different distances ‘D’: 5, 4, 3, 2.5, 2, 1.5, and 1 km. Figure B. Graph showing the evolution of the OR for SES per quantile. The graph has a line per tumor subgroup. The OR for the second and third quantile were not statistically significant different that the OR for the lower quantile, only the OR for the higher quantile was statistically significant. (DOCX) [file pone.0171881.s001.docx]

Supplementary Data, Table A: list of industrial groups, together with their E-PRTR categories, and number of installations by industrial group and autonomous region.

|  |  | Autonomous regions | | | | | |  |
| --- | --- | --- | --- | --- | --- | --- | --- | --- |
| Industrial group | E-PRTR category | Catalonia | Madrid Region | Basque Country | Aragon | Navarre | Other regions^a^ | TOTAL |
| Combustion installations | 1.c | 15 | 5 | 8 | 8 | 3 | 3 | 42 |
| Refineries and coke ovens | 1.a, 1.d | 2 | 0 | 2 | 0 | 0 | 0 | 4 |
| Production and processing of metals | 2.a, 2.b, 2.c.i, 2.c.ii, 2.d, 2.e | 15 | 9 | 70 | 11 | 10 | 4 | 119 |
| Galvanization | 2.c.iii | 5 | 5 | 5 | 1 | 2 | 1 | 19 |
| Surface treatment of metals and plastic | 2.f | 58 | 36 | 49 | 25 | 12 | 17 | 197 |
| Mining industry | 3.a, 3.b | 18 | 8 | 0 | 5 | 6 | 2 | 39 |
| Cement and lime | 3.c, 3.d | 13 | 6 | 5 | 4 | 3 | 2 | 33 |
| Glass and mineral fibers | 3.e, 3.f | 9 | 1 | 3 | 1 | 2 | 4 | 20 |
| Ceramic | 3.g | 39 | 8 | 3 | 15 | 4 | 17 | 86 |
| Organic chemical industry | 4.a | 66 | 7 | 13 | 10 | 5 | 5 | 106 |
| Inorganic chemical industry | 4.b | 20 | 2 | 9 | 14 | 0 | 1 | 46 |
| Fertilizers | 4.c | 7 | 0 | 0 | 2 | 1 | 0 | 10 |
| Biocides | 4.d | 9 | 0 | 0 | 3 | 0 | 0 | 12 |
| Pharmaceutical products | 4.e | 30 | 9 | 0 | 2 | 0 | 0 | 41 |
| Explosives and pyrotechnics | 4.f | 1 | 1 | 4 | 1 | 1 | 1 | 9 |
| Hazardous waste | 5.a, 5.b | 32 | 7 | 11 | 3 | 3 | 4 | 60 |
| Non-hazardous waste | 5.c, 5.d | 36 | 7 | 19 | 12 | 8 | 4 | 86 |
| Disposal or recycling of animal waste | 5.e | 9 | 2 | 3 | 2 | 2 | 0 | 18 |
| Urban waste-water treatment plants | 5.f, 5.g | 24 | 22 | 1 | 2 | 2 | 2 | 53 |
| Paper and wood production | 6.a, 6.b, 6.c | 28 | 2 | 13 | 13 | 6 | 1 | 63 |
| Pre-treatment or dyeing of textiles | 9.a | 7 | 0 | 1 | 0 | 1 | 0 | 9 |
| Tanning of hides and skins | 9.b | 2 | 0 | 0 | 0 | 0 | 0 | 2 |
| Food and beverage sector | 8.a, 8.b, 8.c | 66 | 17 | 7 | 29 | 22 | 4 | 145 |
| Surface treatment using organic solvents | 9.c | 12 | 11 | 12 | 2 | 6 | 7 | 50 |
| Production of carbon or electro-graphite | 9.d | 0 | 0 | 0 | 0 | 1 | 1 | 2 |
| TOTAL |  | 523 | 165 | 238 | 165 | 100 | 80 | 1271 |

^a^These adjacent regions include industries very close to the individuals.

Supplementary Data

Sensitivity analysis to choose the distance for the industrial area by fitting the models for the following distances ‘D’: 5, 4, 3, 2.5, 2, 1.5, and 1 km. There is a change in the trend of urban distance at 2 km with increase in the OR. This increase could be accounting for the industrial pollution at distances of 2km or closer to the industry.

**Figure A**. Estimated OR for industrial exposure and urban exposure with different distances ‘D’: 5, 4, 3, 2.5, 2, 1.5, and 1 km.


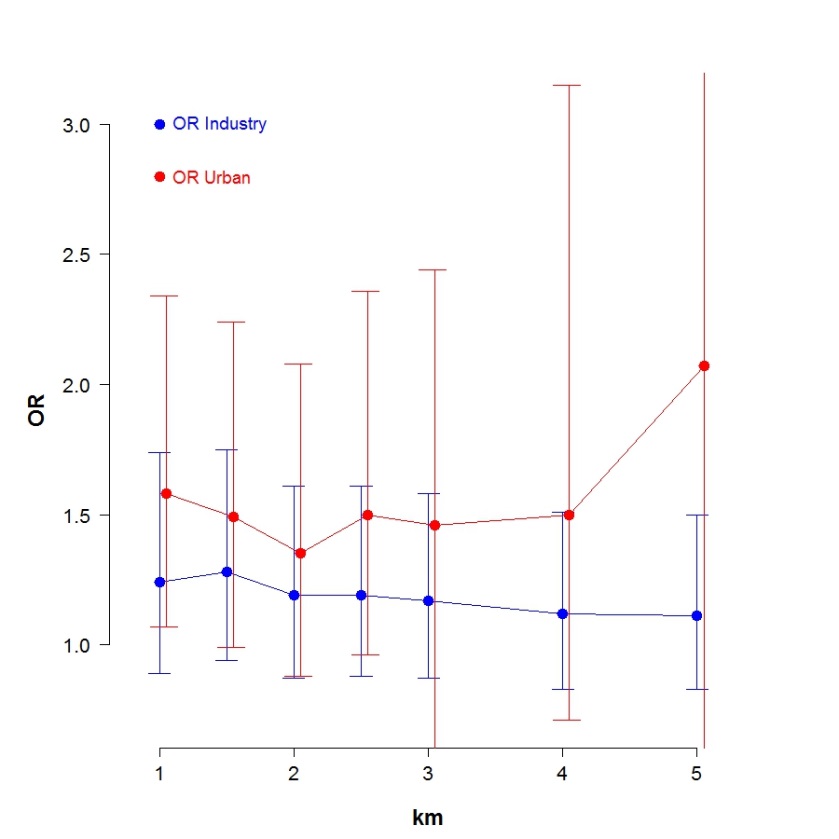


**Figure B**. Graph showing the evolution of the OR for SES per quantile. The graph has a line per tumor subgroup. The OR for the second and third quantile were not statistically significant different that the OR for the lower quantile, only the OR for the higher quantile was statistically significant.


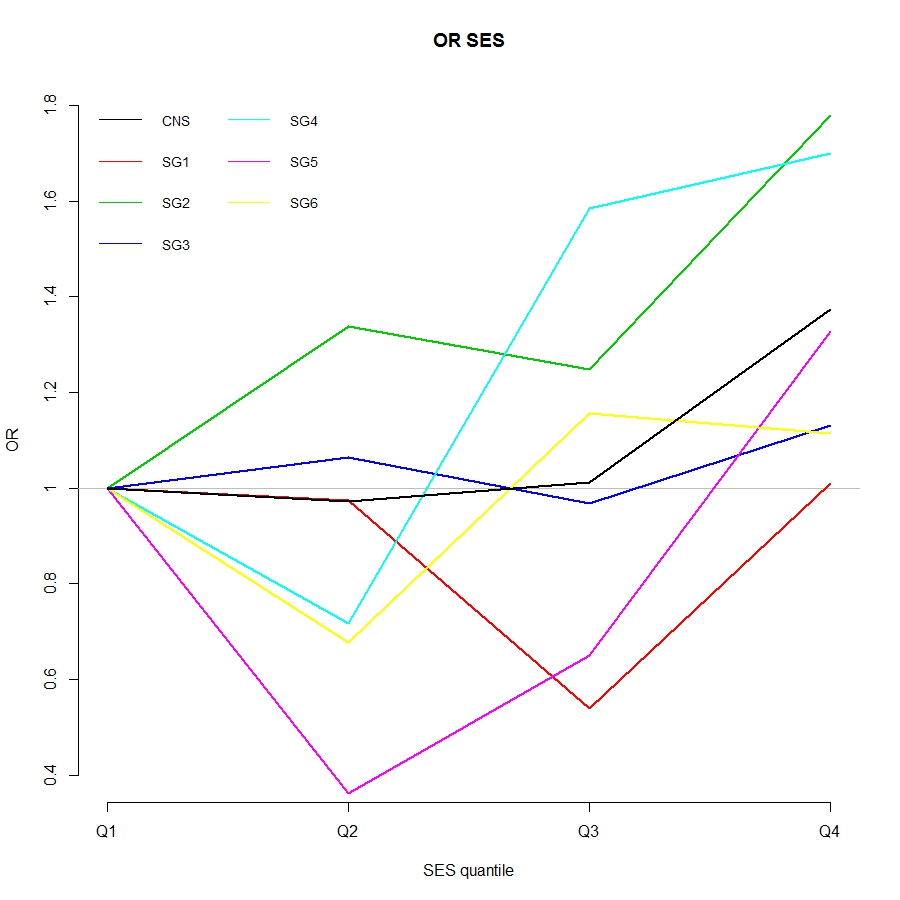


*SG1= Ependymomas and choroid plexus tumor; SG2 = Astrocytomas, SG3 = Intracranial and intraspinal embryonal tumors (IIET); SG4 = Other gliomas; SG5 = Other specified intracranial and intraspinal neoplasms (other specified); SG6 = Unspecified intracranial and intraspinal neoplasms (unspecified)*
